# Whole‐genome SNP markers reveal conservation status, signatures of selection, and introgression in Chinese Laiwu pigs

**DOI:** 10.1111/eva.13124

**Published:** 2020-09-16

**Authors:** Xiaopeng Wang, Hui Zhang, Min Huang, Jianhong Tang, Lijuan Yang, Zhiqiang Yu, Desen Li, Guixin Li, Yongchuang Jiang, Yanxiao Sun, Shudong Wei, Pan Xu, Jun Ren

**Affiliations:** ^1^ Guangdong Laboratory for Lingnan Modern Agriculture College of Animal Science South China Agricultural University Guangzhou China; ^2^ Jinan Conservation Farm for Laiwu Pigs Jinan China; ^3^ School of Animal Science and Technology Jiangsu Agri‐animal Husbandry Vocational College Taizhou China

**Keywords:** introgression, Laiwu pigs, population genetics, selection signatures

## Abstract

Laiwu pigs are a Chinese indigenous breed that is renowned for its exceptionally high intramuscular fat content (average greater than 6%), providing an excellent genetic resource for the genetic improvement of meat quality of modern commercial pigs. To uncover genetic diversity, population structure, signature of selection, and potential exotic introgression in this breed, we sampled 238 Laiwu pigs from a state‐supported conservation population and genotyped these individuals using GeneSeek 80K SNP BeadChip. We then conducted in‐depth population genetics analyses for the Laiwu pig in a context of 1,116 pigs from 42 Eurasian diverse breeds. First, we show that the current Laiwu population has more abundant genetic diversity than the population of 18 years ago likely due to gene flow from European commercial breeds. Both neighbor‐joining (NJ) and principal component analyses indicate the introgression of European haplotypes into Laiwu pigs. The admixture analysis reveals that an average 26.66% of Laiwu genetic components are of European origin. Then, we assigned the tested individuals to different families according to their clustering patterns in the NJ tree and proposed a family‐based conservation strategy to reduce the risk of inbreeding depression in Laiwu pigs. Next, we explored three statistics (ROH and iHS and EigenGWAS) to identify a list of candidate genes for fat deposition, reproduction, and growth in Laiwu pigs. Last, we detected a strong signature of introgression from European pigs into Laiwu pigs at the *GPC6* locus that regulates the growth of developing long bones. Further association analyses indicate that the introgressed *GPC6* haplotype likely contributed to the improvement of growth performance in Laiwu pigs. Altogether, this study not only benefits the better conservation of the Laiwu pig, but also advances our knowledge of the poorly understood effect of human‐mediated introgression on phenotypic traits in Chinese indigenous pigs.

## 1 INTRODUCTION

**FIGURE 1:**
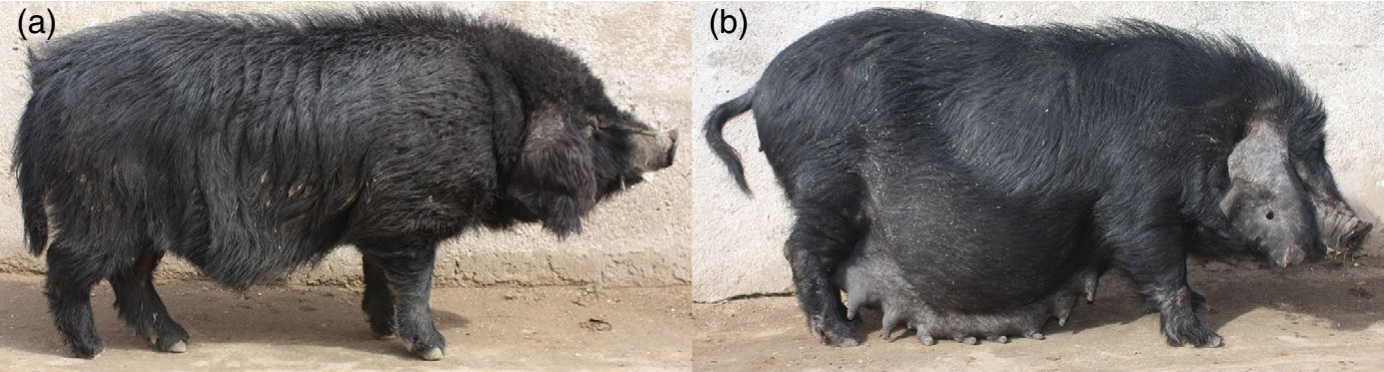
Appearance of Laiwu pigs. (a) Boar. (b) Sow

Laiwu pigs (Figure 1) were originally distributed in Laiwu City, Shandong Province of China. This breed is renowned for its desirable meat quality and exceptionally high intramuscular fat content in pork with an average value of ~6% as compared to less than 2% in European breeds (Chen, Fang, Wang, Wang, & Zeng, 2017). Hence, Laiwu pigs provide excellent genetic materials for the improvement of meat quality in the present‐day pig industry. For this reason, Laiwu pigs have been included in the conservation list of China's livestock and poultry genetic resource by the Ministry of Agriculture of China (Wang et al., 2011).

Archaeological evidence shows that modern and ancient (5,300–6,500 years ago) Laiwu pigs had nearly identical head bones, indicating that Laiwu pigs have a long breeding history of more than 5,000 years. In the early 20th century, Yantai and Qingdao cities of Shandong province were colonized by Britain and Germany, respectively. Western pig breeds such as Berkshire, Yorkshire, and Duroc were then introduced to the Shangdong province to cross with local breeds (Wang et al., 2011). It hence raises the possibility that Shangdong indigenous pigs including the Laiwu pig could have genetic components introgressed from Western breeds. The possibility was supported by our recent study based on whole‐genome SNP markers. We showed that approximately one‐fourth of genetic components in the genome of Laiwu pig are of European origin (Huang et al., 2019). However, the genomic distribution and phenotypic effect of the introgressed European haplotypes in Laiwu pigs remain elusive. In addition, the conservation population of Laiwu pigs is restricted to a single state‐supported farm (Wang et al., 2011). The conservation status including genetic diversity, potential risk of inbreeding depression, and family structure is largely unknown in Laiwu pigs.

In this study, we explored whole‐genome SNP markers to investigate the conservation status, genomic signatures of selection and introgression and their effect on phenotypic traits of all Laiwu pigs (*n* = 238) from the current conservation population in a context of 1,116 individuals from 42 Eurasian diverse breeds. The findings enable us to propose a reliable and sustainable conservation strategy for the Laiwu pig, and provide novel insight into historical contribution of European lineages to Chinese indigenous pigs.

## 2 MATERIALS AND METHODS

### 2.1 Ethics statement

All experimental procedures were approved by the Animal Care and Use Committee of South China Agricultural University, Guangzhou, China.

### 2.2 Sample and genotyping

Ear tissues of 238 Laiwu pigs were sampled from the national conservation farm for Laiwu pigs in Shandong province of China in 2018. These individuals represent the most comprehensive genetic diversity of the Laiwu pig. Genomic DNA was extracted from the ear tissue using a traditional phenol/chloroform method. DNA samples were genotyped for 68,516 SNPs using GeneSeek Genomic Profiler Porcine HD BeadChip (Neogen Corporation, USA) according to the manufacturer's instructions.

To investigate the genetic diversity and population structure of Laiwu pigs in a global perspective, we integrated the 80K SNP data with the Illumina 60K (61,565 SNPs) SNP data of 878 pigs from 35 Chinese pig breeds, one hybrid breed and six European commercial pig breeds (Table S1). The 878 pigs included 18 Laiwu pigs sampled in 1999. Of the 878 pigs, 299 individuals were tested in a previous study (Ai, Huang, & Ren, 2013), 107 individuals were reported in (Wang et al., 2018), and the other 472 individuals were studied in (Xu, Wang, et al., 2019; Xu, Sun, et al., 2019; Yang et al., 2017). We converted the raw data to PLINK v1.9 (Purcell et al., 2007) input files and obtained a common set of 42,464 SNPs from 1,116 individuals. Then, we conducted the following quality control procedures for the merged SNP data using PLINK v1.9 (Purcell et al., 2007): (1) We randomly selected one individual from one pair of highly related animals with an identity‐by‐state score of greater than 0.99 in Laiwu pigs, (2) retained SNPs with minor allele frequencies (MAF) of no less than 0.01, (3) removed SNPs and individuals with call rates of lower than 90%, and (4) discarded all unmapped SNPs and those on sex chromosomes. A final set of 35,027 SNPs from 1,111 pigs were used for the following analyses.

### 2.3 Genetic diversity analyses

Expected heterozygosity (He), observed heterozygosity (Ho), effective population size (Ne), and linkage disequilibrium (LD) decay were calculated to evaluate the genetic diversity of the tested populations, including the current (LWH, *n* = 233) and early‐day conservation population (LWH1, *n* = 18) of Laiwu pigs. He and Ho were calculated using PLINK v1.9 (Purcell et al., 2007) with default setting. Pair‐wise LD was evaluated by the correlation coefficient (*r* ^2^) between alleles at two separate SNP loci using PLINK v1.9 (Purcell et al., 2007) under the default setting. According to a previous study (Sved, 1971), Ne was estimated based on the equation Ne_t_ = (1/4c)(1/*r* ^2^ − 1), where Ne_t_ is the effective population size of t generations age and is calculated as t = 1/2c, *r* ^2^ is the LD between pair‐wise SNPs, and c is the genetic distance in Morgan between a pair of SNPs (Tortereau et al., 2012).

### 2.4 Inbreeding coefficient

Two measures of genomic inbreeding coefficient were calculated for each population using PLINK v1.9 (Purcell et al., 2007). (1) SNP‐based inbreeding coefficient (*F*) that was calculated using 25,839 SNPs with pair‐wise LD values of less than 0.5, and the command was set as “‐‐indep‐pairwise 50 10 0.5.” (2) Runs of homozygosity (ROH)‐based inbreeding coefficient (*F*_ROH_) that was measured by the ratio of the total length of ROH to the length of autosomes (2.45 Gb in this study; Mcquillan et al., 2008). ROHs were identified for each individual using PLINK v 1.9 (Purcell et al., 2007) with the following parameters (Shi et al., 2020; Xie et al., 2019): (a) The minimum number of SNPs in a sliding window was 50; (b) the minimum ROH length was set to 1 Mb to eliminate the impact of strong linkage disequilibrium; (c) each ROH need contain a minimum of 80 consecutive SNPs, which was calculated by following equation (Lencz et al., 2007), $l=\log_{e}\left(\alpha /\left(n_{s}\times n_{i}\right)\right)/\log_{e}\left(1‐het\right)$ where *α* is the percentage of false‐positive ROH (set to 0.05 in this study), *n_s_* is the number of SNPs per individual, *n_i_* is the number of individuals, and *het* is the proportion of heterozygosity across all SNPs. (d) One heterozygous and five missing calls per window were allowed to avoid false negatives caused by occasional genotyping errors or missing genotypes. (e) The minimum SNP density was 1 SNP every 100 kb, and the maximum gap between consecutive SNPs was set to 1 Mb. Three ROH estimates were calculated: ROH < 5 Mb, 5 < ROH <10 Mb, and ROH > 10 Mb, an indicative of ancestral (more than 10 generations), middle (5 to 10 generations), and recent (within 5 generations) inbreeding, respectively (Mastrangelo et al., 2016).

### 2.5 Population structure

Two matrices of identical‐by‐state and genetic differentiation *F*_ST_ (Weir & Cockerham, 1984) were calculated for pair‐wise individuals and populations (breeds) via PLINK v1.9 (Purcell et al., 2007). The two matrices were then explored to construct neighbor‐joining (NJ) trees of individuals and populations using PHYLIP v3.69 (Felsenstein, 1989). The NJ trees were visualized via Figtree v1.4.2 (http://tree.bio.ed.ac.uk/software/figtree/). Principal component analysis (PCA) was performed using the GCTA software (Yang, Lee, Goddard, & Visscher, 2011), and the first two PCs were plotted via in‐house R scripts. The ADMIXTURE v1.30 software (Alexander, Novembre, & Lange, 2009) was employed to infer the proportion of introgressed ancestry in the tested populations. To reduce the effect of ascertainment bias, we used the genotype data of the pruned 25,839 SNPs with LD (*r* ^2^) values of less than 0.5. To avoid sampling bias, we randomly selected 10 pigs from each population to calculate the ancestral lineage compositions. Considering the large sample size and heterogenous genetic background of current Laiwu pigs, we classified the 233 Laiwu pigs to 23 groups (each group contained ten pigs, except the last group with 13 pigs) with other random sampling breeds to conduct the Admixture analyses and obtained well‐consistent results (data not shown). The optimal number was determined by cross‐validation error. Finally, the inferred population structure for one of group was visualized using in‐house R scripts.

### 2.6 Signature of selection

Three methods were performed, including ROH occurrences, iHS statistic within LWH2 population, and EigenGWAS between LWH2 and other 34 Chinese local pig breeds. We selected 150 Laiwu pigs (LWH2 pigs) that had Chinese genetic components of greater than the average value (73.25%) of the whole population in the Admixture analysis. We explored 46,955 qualified SNPs from the 150 pigs to conduct the ROH analysis as mentioned above, and a minimum of 60 consecutive SNPs in each ROH. Then, we counted the occurrence times of a given SNP in the identified ROHs of the 150 LWH2 pigs and presented a Manhattan plot of all tested SNPs against their positions in autosomes. We defined the most frequently observed SNPs in ROHs at the top 1% (empirical distribution) level as significant loci putatively under selection (Pemberton et al., 2012). In addition, we used the integrated haplotype score (iHS; Voight, Kudaravalli, Wen, & Pritchard, 2006) to detect signatures of selection in the 150 Laiwu pigs using the *selscan* v1.2.0 a software under default parameters (Szpiech & Hernandez, 2014). We have matched 42,517 out of 46,955 SNPs with the ancestral and derived alleles obtained from the previous study (Bianco, Nevado, Ramos‐Onsins, & Pérez‐Enciso, 2015), and the rest 4,438 SNPs were kept the original status. BEAGLE v4.0 (Browning & Browning, 2007) was employed to phase haplotypes and impute missing genotypes. The unstandardized iHS scores were then normalized through the *norm* software with default parameters (Szpiech & Hernandez, 2014). The |iHS| values were plotted as a Manhattan figure, and those at the top 1% of empirical distribution were defined as potentially selected regions (100 kb).

To identify additional candidate genes under selection in Laiwu pigs, we conducted a genome‐wide association study (GWAS) to identify candidate loci under selection. This pattern used the eigenvector (the first principal component, PC1) from the PCA as a “phenotype” (EigenGWAS; Chen, Lee, Zhu, Benyamin, & Robinson, 2016) for 35,027 qualified SNPs between the 150 LWH2 and 683 individuals from 34 other Chinese pig breeds. The EigenGWAS was supported by the fact that PC1 clearly separated the LWH2 individuals from the other Chinese indigenous pigs (Figure S1). SNPs with the Bonferroni corrected *p* values of less than 10^–6^ (0.05/35027) were considered the significant loci. *F*_ST_ statistic was calculated to validate the veracity of EigenGWAS analysis with PC1 as the phenotype. We also calculated Pearson's correlation coefficient between the EigenGWAS and *F*_ST_ statistics using in‐house R scripts. The selection signatures detected by at least two statistics were considered candidate loci under selection.

### 2.7 Functional annotation of candidate genes

Candidate genes were identified at 100 kb regions (upstream and downstream 50 kb) flanking these candidate loci via the *Ensembl* database (*Sscrofa* 11.1; http://asia.ensembl.org/index.html). The Gene Ontology (GO) and Kyoto Encyclopedia of Genes and Genomes (KEGG) pathway were analyzed for the functional enrichment of the retrieved candidate genes via the Matescape database (Tripathi et al., 2015).

### 2.8 Detection of introgression

The *f3* test (David, Kumarasamy, Nick, Price, & Lalji, 2009) was performed to investigate the significance of admixture in Laiwu pigs using TreeMix software (Pickrell & Pritchard, 2012) with the default setting. The *f3* test with the form of (A; B, C), an extreme negative *f3* value indicates that significant gene flow to population A from populations B and C. Z‐scores were calculated, and the Z‐score less than −2 was defined as significant. The *qpAdm* statistic (Patterson et al., 2012) was preformed to estimate the ancestry proportion of LWH pigs, using the 11 Chinese indigenous pig breeds (EHL, JH, DX, GX, TC, SZL, DS, LUC, CJX, LTT, and RC, defined as CNref) and 6 European pigs (DRC, LW, LR, PIT, USBK, and USHS, defined as EUref) as reference populations based on the result of *f3*, Chinese wild boar (WB) and 10 *Sus verrucosus* (SVSV) individuals (Yang et al., 2017) as outgroups.

Patterson's D statistic (Green et al., 2010) was employed to assess the introgression from European pig breeds to LWH2 population using the qualified autosomes SNP makers (35,027 SNPs). In the tree topology (((P1, P2), P3), O), 10 *Sus verrucosus* (SVSV) individuals as outgroup (O; Yang et al., 2017) were used to test whether the P1 (Chinese wild boars) and P2 (LWH2) shared more alleles with a candidate introgressor—P3 (EU), including six European modern pig breeds. To quantify the size of the introgression, the *f_dM_* devised by Malinsky (Malinsky et al., 2015) was calculated, using a 10 SNPs sliding window with 2 SNPs stepping (Wang et al., 2020). Positive values of the *f_dM_* stand for introgression between P3 and P2 and negative values for introgression between P3 and P1. *P* values then estimated by Z‐transformed *f_dM_* values assuming the standard normal distribution, and windows with *p* < .01 (*f_dM_* = 0.611) were identified as significantly introgressed genomic regions. Moreover, absolute divergence (*dxy*) and nucleotide diversity (π) in genome‐wide were calculated with the python script from https://github.com/gibert‐Fab/ABBA‐BABA (Martin, Davey, & Jiggins, 2015), using a 10 SNPs sliding window with 2 SNPs stepping. We also used fastPHASE (Scheet & Stephens, 2006) to construct haplotypes using 21 SNPs at the significant selection and introgression gene *GPC6* (11:62,436,143–63,560,908) in tested populations. Finally, we downloaded the resequencing data of 259 Eurasian pigs (including six Laiwu pigs) from previous studies (Chen et al., 2020) and retrieved the *GPC6* region (13,899 qualified SNPs) to perform NJ tree analysis.

### 2.9 Association analyses

To examine the putative effect of the introgression region, we genotyped 365 pigs from the Sujiang breed that was derived from a cross between Chinese Jiangquhai pigs and European Duroc pigs using the GeneSeek Genomic Profiler Porcine HD BeadChip (Xu et al., 2020). A total of 21 qualified SNPs extracted from *GPC6* region on pig chromosome 11. The linear mixed model was fit to this data using GEMMA software (Zhou & Stephens, 2012) to test for the significant effect of SNPs on body weight, body length, and chest circumference. The statistical model was described as following: y=Wα+xβ+u+∈, where *y* is the phenotype; *W* is a matrix of covariates (i.e., fixed effects that contain batch, age and a column of 1s); α is a vector of corresponding coefficients that includes the intercept; x is a vector of SNP genotypes; β is the effect size of SNPs; u is a vector of random polygenic effects with a covariance structure that follows a normal distribution u ∼ *N*(0, K × Vg), where K is a genomic relationship matrix derived from independent SNPs and Vg is the polygenic additive variance, and ε is a vector of random errors.

## 3 RESULTS

### 3.1 Genetic diversity of Laiwu pigs

The current conservation population of Laiwu pigs had the greatest value of He (0.284) and the third greatest value of Ho (0.281) among 35 Chinese indigenous pig breeds tested in this study. In contrast, the early‐day Laiwu population (LWH1) had the third least values of Ho (0.15) and He (0.15) among these 35 breeds (Table S1). The SNP‐based (*F*) and ROH‐based (*F*_ROH_) inbreeding coefficients showed positive correlation (*r* = 0.513, *p* < .001). We subdivided *F*_ROH_ values into three categories, that is, those derived from ROHs of less than 5 Mb (*F*_ROH 0‐5Mb_), from 5 to 10 Mb (*F*_ROH 5‐10Mb_), and greater than 10 Mb (*F*_ROH >10Mb_; Table S2). We found that *F* had significantly positive correlation (*r* = 0.553, *p* < .001) with *F*_ROH >10Mb_. Moreover, ROHs of greater than 10 Mb accounted for nearly half of total ROHs in both LWH1 (47.92%), LWH2 (45.87%), and LWH (45.25%) populations. This indicates that recent inbreeding events within the last five generations occurred in Laiwu pigs. LWH1 pigs had higher values of *F*_ROH_ (0.185) and *F* (0.308) than LWH individuals (*F*_ROH_ = 0.133, *F* = 0.185), which is consistent with the abovementioned results of heterozygosity. The genetic diversity indexes of LWH2 in the medium levels (*F*_ROH_ = 0.164, *F* = 0.270) between LWH1 and LWH pigs (Table S2).

LD extent in each population was estimated as the physical genomic distance at which the genotypic association (*r* ^2^) was 0.3 (Ai et al., 2013). LWH1 pigs had the longer of LD extent ($r_{0.3}^{2}$ = 195.11 kb) among Chinese pigs. The LD value was even greater than those of European pigs from Large White, Landrace, Duroc, and Hampshire breeds, and was more than three and two times than that of LWH pigs ($r_{0.3}^{2}$ = 62.14 kb) and LWH2 population ($r_{0.3}^{2}$ = 79.31 kb). The average effective population size (Ne) of each population was estimated using the method as previously described (Herrero‐Medrano et al., 2013). To reduce potential sampling bias, we calculated Ne for the past five generations. Ne ranged from 84 in Ganxi pigs to 241 in LWH pigs. The Ne of LWH was twice as much as that of LWH1 (Ne = 120) and was roughly equal to the actual number of the LWH population (No = 238). Surprisingly, the Ne of LWH2 (Ne = 271) greater than whole LWH pigs, we speculated some individuals with large LD were removed in LWH2 population (Table S1).

### 3.2 Population structure of Laiwu pigs

#### 3.2.1 | NJ tree

**FIGURE 2:**
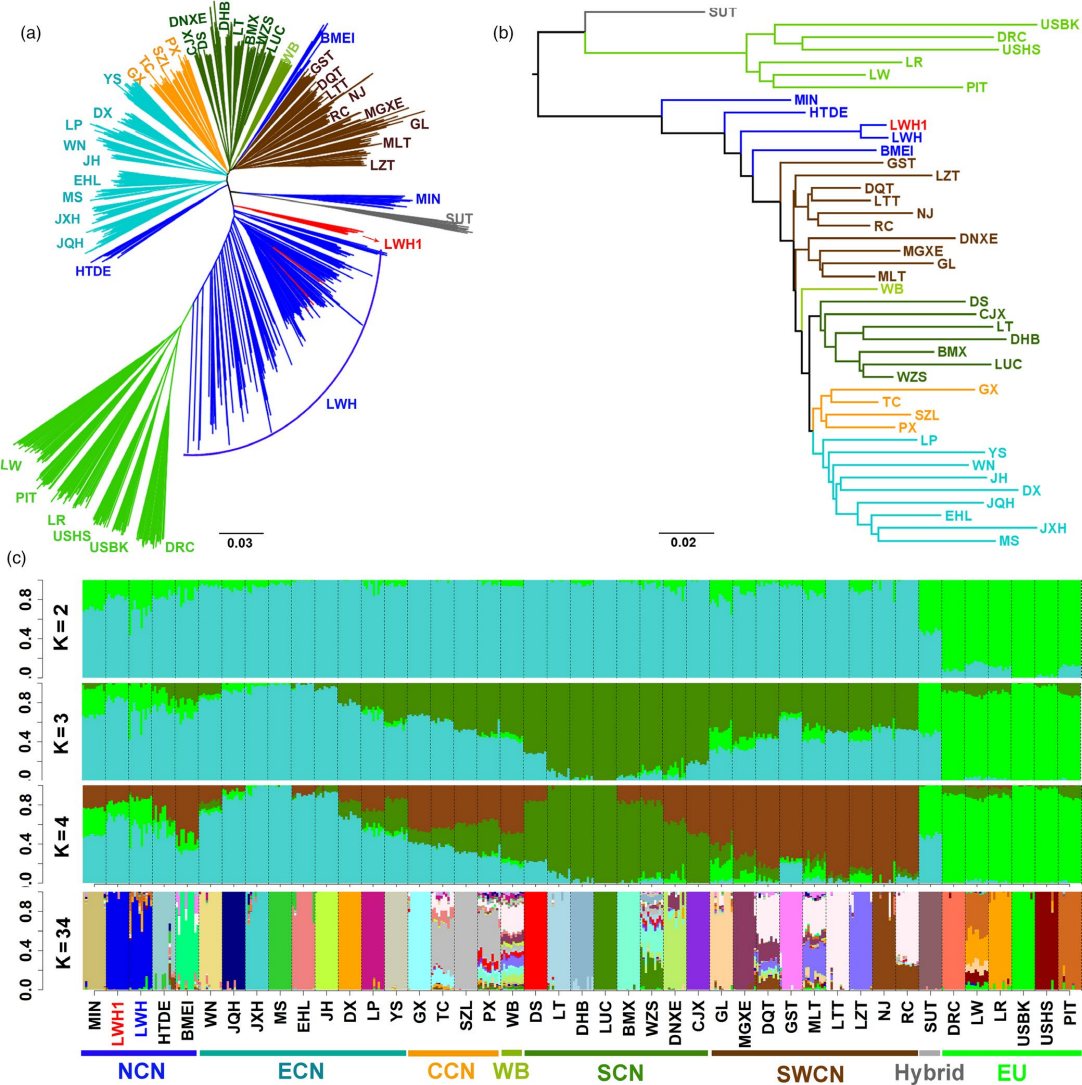
Phylogenetic relationship and population structure of Eurasian pig breeds and wild boars tested in this study. (a) Neighbor‐joining phylogenetic tree of 1,111 pigs from 42 Eurasian breeds and Chinese wild boars. (b) *F_ST_* estimate‐derived dendrogram of 42 Eurasian breeds and Chinese wild boars. (c) The ancestry compositions of 42 Eurasian breeds and Chinese wild boars that were uncovered by ADMIXTURE with the assumed number of ancestries from 2 to 4 and 34. Each color represents one ancestral cluster. Breeds are separated by dotted lines. Laiwu pigs are highlighted by colored axes. Abbreviations of all breeds and wild boars are given in Table S1

First, we conducted an IBS‐derived NJ tree for all tested individuals (Figure 2a). Consisted with our previous reports (Ai et al., 2013; Huang et al., 2019), Eurasian pigs were mainly clustered into two separate clades: European and Chinese clades. We noted that Laiwu (LWH and LWH1) pigs were located at intermediate positions between the two major clades. Multiple LWH individuals did not cluster together and showed close relationships with European modern breeds (Figure 2a). Then, we constructed an *F_ST_*‐based NJ tree for all tested populations (Figure 2b). The two Laiwu populations clustered in one clade and showed the lowest differentiation coefficient (*F_ST_* = 0.02) among all tested populations. Laiwu and other Chinese pigs defined a major branch separating from European pigs in the NJ tree. Among Chinese pigs, Laiwu pigs had closer genetic relationships with pigs from Northern Chinese breeds including Min, Baimei, and Hetao. These breeds gathered together in a paraphyletic pattern, which is most likely caused by gene flow between European modern breeds and Northern Chinese breeds including Laiwu pigs as we previously reported (Wang et al., 2018).

#### 3.2.1 Principal component analysis

European modern breeds, European‐Chinese hybrid breed (Sutai), Laiwu pigs, and other Chinese breeds formed four groupings in the PCA plots (Figure S1). PC1 accounted for 19.9% variance between Chinese and European pigs, and PC2 explained 4.8% variance between Laiwu and other Eurasia pigs. The two Laiwu populations (LWH1 and LWH) clustered together, and a number of LWH individuals showed close relationships with European modern pigs (Figure S1), which is consistent with the NJ clustering pattern.

#### 3.2.2 ADMIXTURE

The ancestral lineage compositions of 430 pigs from global populations are shown in Figure 2c. The K value represents the number of ancestries. As we previously reported (Ai et al., 2013; Huang et al., 2019; Wang et al., 2018), Chinese pigs had three main ancestral lineages, i.e. East Chinese (ECN), South Chinese (SCN), and Southwest Chinese (SWCN) lineages (K = 4). Both LWH and LWH1 individuals showed obvious signatures of introgression with European modern pigs (K = 2, 3, and 4). When K = 4, Laiwu pigs had genetic components of ECN, SWCN, and European origin, and LWH pigs had more ancestral fractions of European lineages. When K = 34 that represented the optimal number of assumed ancestors by cross‐validation error test (Figure S2), a certain proportion of European ancestries were still evidenced in LWH pigs.

### 3.3 Population structure and subfamilies in Laiwu pigs

**FIGURE 3:**
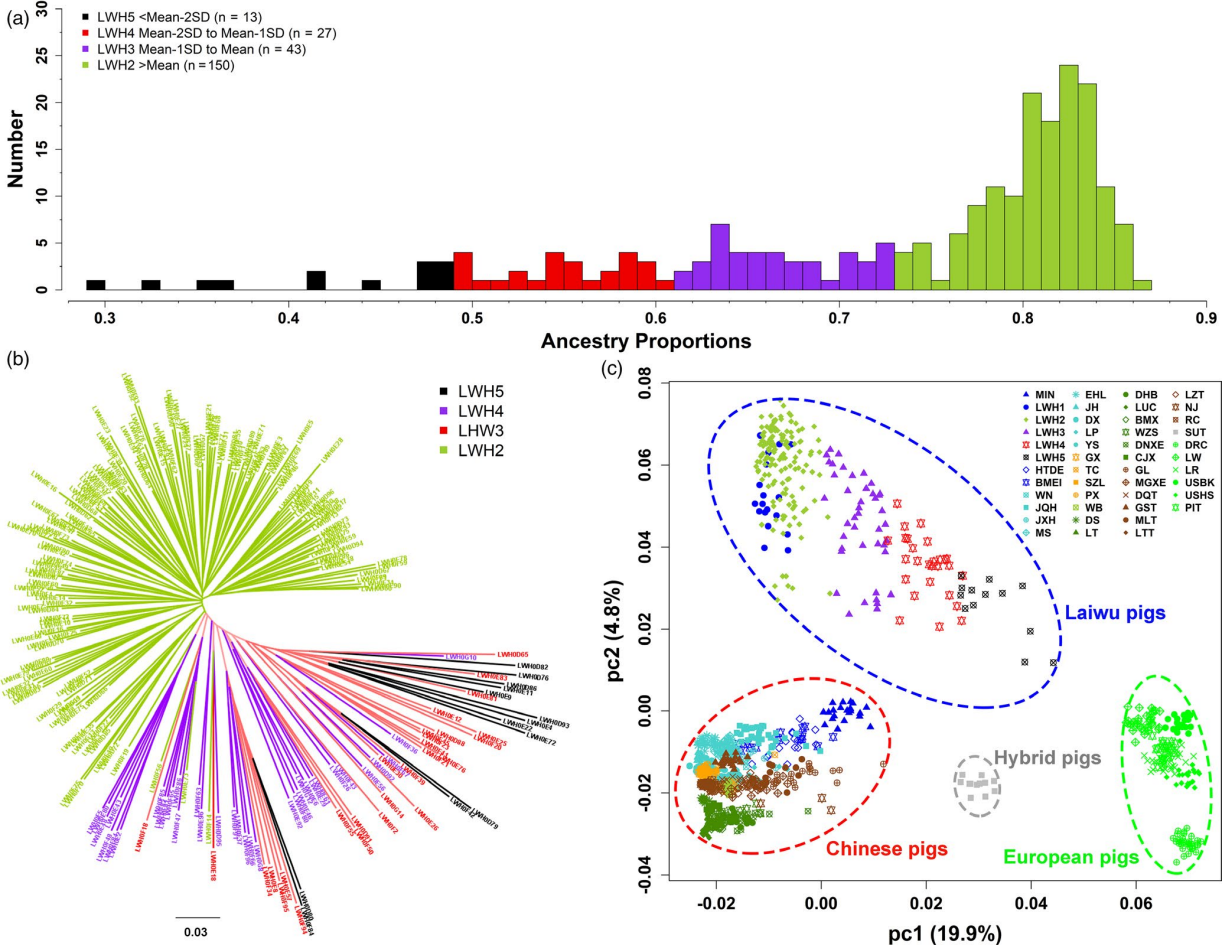
Compositions of Chinese lineages in Laiwu pigs. (a) Distribution of Chinese lineage compositions in 233 Laiwu pigs. Proportions of Chinese lineage in the genomes of Laiwu pigs are shown in the x‐axis. (b) Neighbor‐joining phylogenetic tree for the 233 Laiwu pigs. (c) Principle component analysis of 1,111 pigs including the 233 Laiwu pigs. LWH2, 150 Laiwu pigs with Chinese lineage compositions greater than the mean value of the 233 Laiwu pigs. LWH3, 43 Laiwu pigs with Chinese lineage compositions between the mean value and mean minus one standard deviation (Mean ‐ 1SD). LWH4, 27 Laiwu pigs with Chinese lineage compositions ranging from Mean ‐ 1SD to Mean ‐ 2SD. LWH5, 13 Laiwu pigs with Chinese lineage compositions less than Mean ‐ 2SD. The four populations are indicated by different colors

We made a close examination on the ancestral lineage compositions of all 233 tested Laiwu pigs using the ADMIXTURE analysis (Alexander et al., 2009). When K = 2, a proportion of Chinese lineage ranging from 29.10% to 86.64% was observed in these 233 Laiwu pigs with an average value of 73.25% and a standard deviation (*SD*) of 12.22% (Table S3). We divided the proportion values into four categories, including those greater than the mean value (≥73.25%, LWH2), between the mean and the mean minus one *SD* (61.03% to 73.25%, LWH3), between the mean minus two SDs and the mean minus one *SD* (48.81% to 61.03%, LWH4), and less than the mean minus two SDs (< 48.81%, LWH5). The four categories contained 150 (LWH2), 43 (LWH3), 27 (LWH4), and 13 (LWH5) individuals, respectively (Figure 3a). We propose that the 150 LWH2 individuals should be used as the nucleus conservation population, and the 70 LWH3 and LWH4 individuals can be treated as a candidate conservation population. The 13 LWH5 individuals should be discarded from the conservation population due to their unusually low proportions (<48.81%) of Chinese lineage, remarkable genetic differentiation from LWH2 pigs (Figure 3b), and close relationships with European modern breeds (Figure 3c).

**FIGURE 4:**
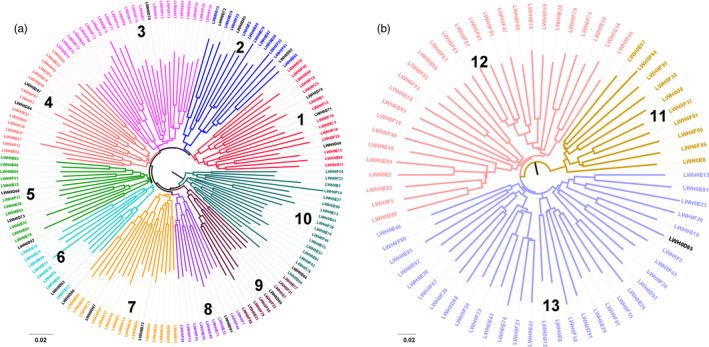
Family classification of Laiwu pigs. (a) 150 LWH2 pigs are assigned to ten families in the neighbor‐joining (NJ) phylogenetic tree. (b) 70 LWH3 and LWH4 individuals are classified into three families in the NJ tree. Boars are indicated in black. Definitions of LWH2, LWH3, and LWH4 pigs are given in the legend of Figure 3

We further reconstructed the IBS‐based NJ trees for LWH2, LWH3, and LWH4 individuals (Figure 4). According to the clustering pattern in the NJ tree, the 150 LWH2 pigs pertained to ten families (branches), each of which contained at least one boar and variable number (9 to 24) of sows (Figure 4a). The 70 LWH3 and LWH4 pigs belonged to three families (branches), and only one family had one boar (Figure 4b). Finally, we calculated the ROH and *F* values for the 220 individuals from the LWH2, LWH3, and LWH4 populations (Figure S3). We highlighted nine and two individuals as the outliers, because these individuals had the ROH and *F* values of greater than the mean value plus two standard deviations. Hence, special attention should be paid to these highly inbred individuals to avoid inbreeding depression. As expected, the LWH3 and LWH4 individuals had lower *F*_ROH_ (0.076) and *F* (0.045) values than LWH2 pigs (*F*_ROH_ = 0.164; *F* = 0.270), which is attributable to a high proportion of European lineage in the LWH3 and LWH4 pigs (Table S3).

### 3.4 Candidate genes under selection in Laiwu pigs

#### 3.4.1 Runs of homozygosity

**FIGURE 5:**
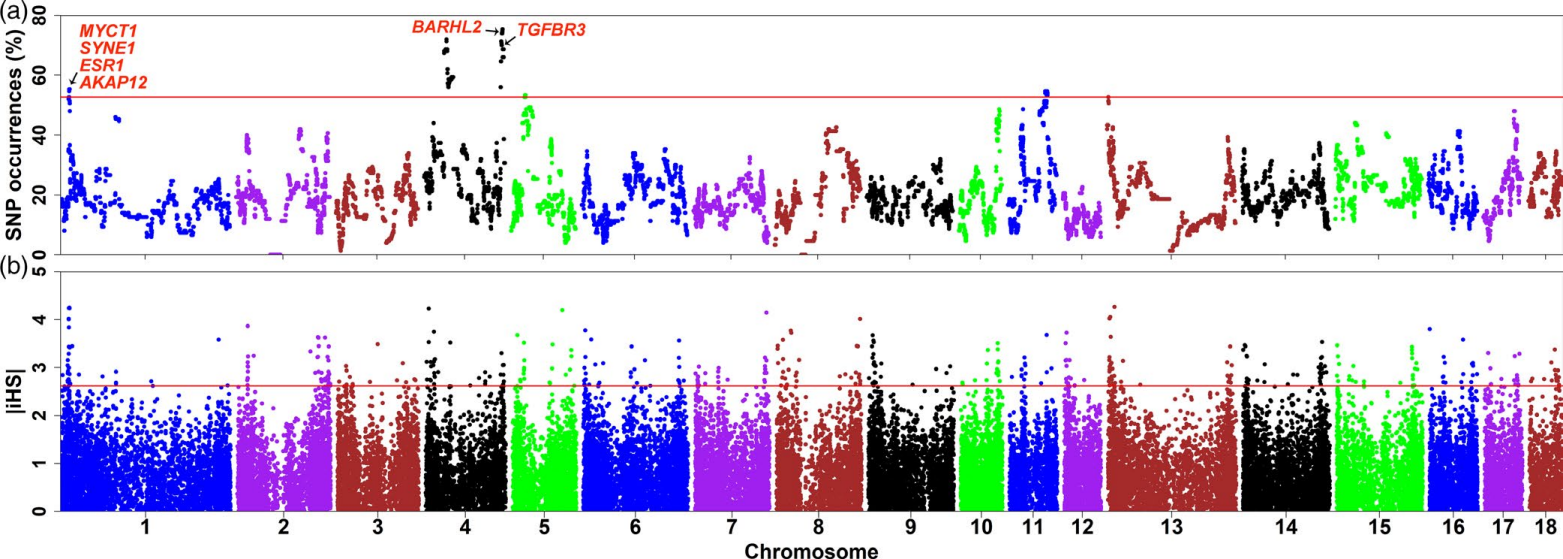
Signatures of selection in the genomes of Laiwu pigs. (a) Manhattan plot of incidence of each SNP in runs of homozygosity (ROH). (b) Genome‐wide distribution of selection signatures revealed by the iHS statistic. The read lines represent the top 1% threshold

We found that 0.39% of SNPs not occur in any ROHs of Laiwu pigs, and 2 chromosomes had long non‐ROH cold‐spot regions: SS2 (55.655–71.799 Mb) and SSC8:43.027–50.000 Mb). The cold‐spot regions could be resulted from high recombination rate, or selection for heterozygous advantage (Upadhyay et al., 2017; Xu, Sun, et al., 2019). On the other hand, ROHs of variable lengths were distributed across the genomes of Laiwu pigs. The most frequently observed ROH was located within a region of 0.16 Mb on SSC4. We defined the top 1% of the most frequent ROHs as candidate regions under selection (Figure 5a), leaving us 480 SNPs on five chromosomes as candidate loci (Table S4).

#### 3.4.2 iHS

The greatest value |iHS| values on SSC1, and the top 1% SNPs (452 SNPs; Table S4) were considered candidate loci under selection (Figure 5b). We observed a significantly positive correlation coefficient between the ROH and iHS statistics (*r* = 0.071, *p* < 10^–16^). This supports our assumption that the significant ROH regions are not only due to demography but also positive selection (Xu, Sun, et al., 2019; Zhang, Guldbrandtsen, Bosse, Lund, & Sahana, 2015).

#### 3.4.3 EigenGWAS

**FIGURE 6:**
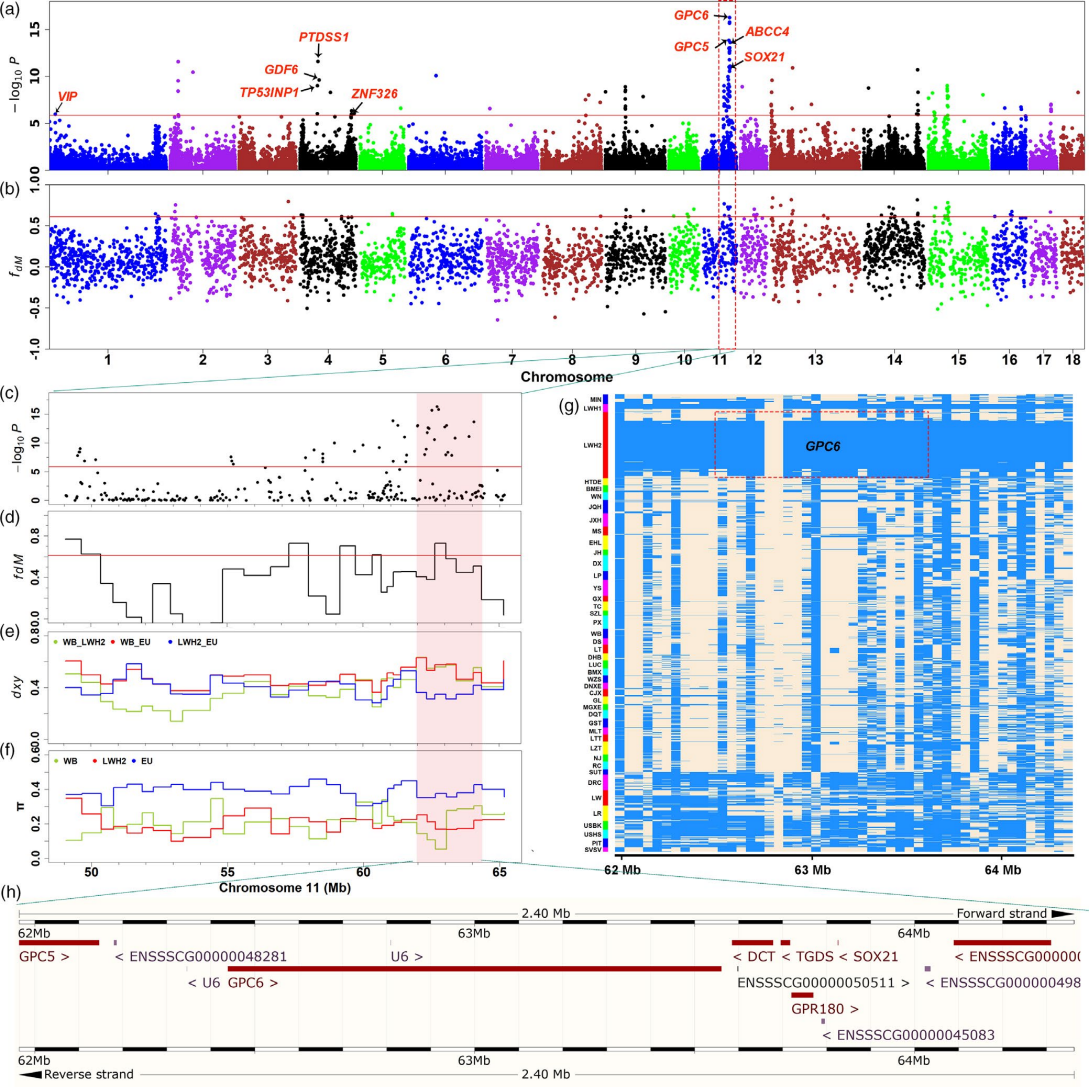
Signatures of selection and introgression revealed by comparison of the genomes of Laiwu pigs with those of other Chinese indigenous pigs. (a) Genome‐wide analysis of EigenGWAS between 150 Laiwu pigs and other 683 Chinese pigs. The red line indicates the 0.05/35027 threshold. The y‐axis represents the negative logarithm of *P* values for the EigenGWAS that explored the first principle component as phenotype. (b) Introgression regions identified in the LWH2 genome. A modified *f*‐statistic (*f_dM_*) for 10 SNPs per window with 2 SNPs stepping is plotted along the autosomes. The red line corresponds to significance level threshold (*p* = .01). (c–f) The 16 Mb region in SSC11 (49061964–65169959) detected by EigenGWAS, *f_dM_*, absolute divergence (*dxy*) and nucleotide diversity (π) statistics, respectively. The area (11:61964048–64362534) on the pink background showed the significant selection and introgression, low *dxy* value (blue line) between LWH2 and EU, and reduced the π value (red line) in LHW2. (g) Haplotype heat map of the 2.4 Mb (11:61964048–64362534) region encompassing the *GPC6* gene. Major and minor alleles in Laiwu pigs are indicated by beige and light blue, respectively. (h) Annotated genes in the region of 2.4 Mb in SSC11 (61964048–64362534)

In the EigenGWAS analysis, we identified 110 SNPs surpassing the genome‐wide significant threshold (0.05/35027; Figure 6a, Table S4). The *F*_ST_ and EigenGWAS statistics showed significant positive correlation (*r* = 0.91, *p* < 2.2 × 10^–16^). Of note, the top SNP located in a large region (47.87–64.05 Mb) on SSC11.

Moreover, 32 significant SNPs were detected by at least two methods of ROH, iHS, and EigenGWAS statistics (Figure S4a, Table S4), and 24 candidate genes resided in 100 kb regions surrounding the 32 SNPs. These candidate genes were significantly enriched in 5 GO terms (Figure S5a, Table S5). A number (14/24) of genes were involved in growth‐related biological processes, such as “muscle cell proliferation (GO:0033002),” bone development “BMP signaling pathway (GO:0030509),” and “regulation of animal organ morphogenesis (GO:2000027)”; growth‐related candidate genes including *MYCT1*, *SYNE1*, *PTDSS1*, *GDF6*, *BARHL2*, *ZNF326*, *GPC5*, *GPC6*, *SOX21,* and *ABCC4* (Table 1); fatness‐related candidate genes including *ESR1*, *TP53INP1,* and *GPC6*; and reproduction‐related candidate genes including *VIP*, *ESR1*, *AKAP12,* and *TGFBR3* (Table 1). A total of 333 quantitative trait loci (QTL) have been reported around these regions according to the pig QTL database (https://www.animalgenome.org/cgi‐bin/QTLdb/SS/index), and 66.97% are related to meat and carcass traits, 13.81% are associated with health traits, 13.21% are related to production traits, and 4.2% are associated with reproduction (Table S6). It is worth mentioning that only two significant SNPs on SSC4 were detected by the three methods (Figure S4a). One SNP locates at 5.8 kb downstream of the *ZNF326* gene, and the other SNP is in an intron of the *GDF6* gene.

**TABLE 1:** Candidate genes under selection in Laiwu pigs

Position (Mb)	Gene	Method	Phenotype	Reference
1:13.61‐13.62	*VIP*	^b^, ^c^	Reproduction	Lacombe et al. (2007)
1:13.65‐13.69	*MYCT1*	^a^, ^b^	Growth and fatness	Winkler et al. (2015)
1:14.03‐14.20	*SYNE1*	^a^, ^b^	Growth and weight	Jianlin et al. (2010)
1:14.22‐14.49	*ESR1*	^a^, ^b^	Fat deposition and litter size	Ohlsson et al. (2000) Muñoz et al. (2007)
1:14.91‐15.02	*AKAP12*	^a^, ^b^	Reproduction and feed conversion ratio	Akakura et al. (2008), Messad et al. (2019)
4:40.30‐40.38	*PTDSS1*	^a^, ^c^	Skeletal dysplasia	Piard et al. (2018)
4:40.47‐40.49	*GDF6*	^a^, ^b^, ^c^	Skeletal development	Clendenning and Mortlock (2012)
4:41.75‐41.77	*TP53INP1*	^a^, ^c^	Fatness	Seillier et al. (2015)
4:125.10‐125.32	*TGFBR3*	^a^, ^b^	Embryonic viability	Chermula et al. (2018)
4:126.03‐126.03	*BARHL2*	^a^, ^b^	Body height	Allen et al. (2010)
4:126.68‐126.72	*ZNF326*	^a^, ^b^, ^c^	Body height	Allen et al. (2010)
11:60.79‐62.15	*GPC5*	^a^, ^b^, ^c^	Body height	Wood et al. (2014)
11:62.44‐63.56	*GPC6*	^a^, ^c^	Skeletal development, fat deposition	Capurro et al. (2017), Ding et al. (2019)
11:63.87‐63.83	*SOX21*	^a^, ^c^	Growth and height	Cheung et al. (2017)
11:64.09‐64.31	*ABCC4*	^a^, ^c^	Body height	Fox et al. (2007)

^a^ ROH.

^b^ iHS.

^c^ EigenGWAS.

### 3.5 Detection of introgression

There were 40 extreme Z‐scores of the *f3* test indicated that LWH pigs were significantly admixed by LWH1, eleven Chinese pig breeds (CNref), six European commercial pig breeds (EUref; Table S7). The result of *qpAdm* revealed that the LWH pigs had ancestry proportions of CNref (74.2%) and EUref (25.8%), that consistent with the ADMIXTURE result. The significant positive D value showed clear evidence from the genome‐wide level that introgression from EU to LWH2 ((((WB, LWH2), EU), OUT), D = 0.198, *p* < 2 × 10^–16^ (*t* test)). A total of 58 significant introgression regions have been detected by *f_dM_*‐statistic in 11 chromosomes (Figure 6b, Table S8). The GO and KEGG analyses of the genes introgressed from EU showed that terms related to growth (“regulation of developmental growth (GO:0048638),” “developmental cell growth (GO:0048588),” “developmental growth (GO:0048589)” et al), metabolism (“Fatty acid metabolism (hsa01212),” “fatty acid oxidation (GO:0019395),” “regulation of protein catabolic process (GO:0042176),” “carbohydrate transport (GO:0008643)”) and environment adaptation “positive regulation of cold‐induced thermogenesis (GO:0120162)” were largely enriched (Figure S5b, Table S5). Calculation of the *f_dM_* of 233 LWH pigs using the tree topology (((WB, LWH), EU), OUT) demonstrated that the introgressed regions (*f_dM_* > 0) included 27.44% of the LWH genome. We then compared the average nucleotide diversity value (π) of those admixed regions in LWH (π = 0.289) and LWH1 (π = 0.231). The result showed that 25.11% nucleotide diversity was boosted by the European introgression in current Laiwu pigs.

**FIGURE 7:**
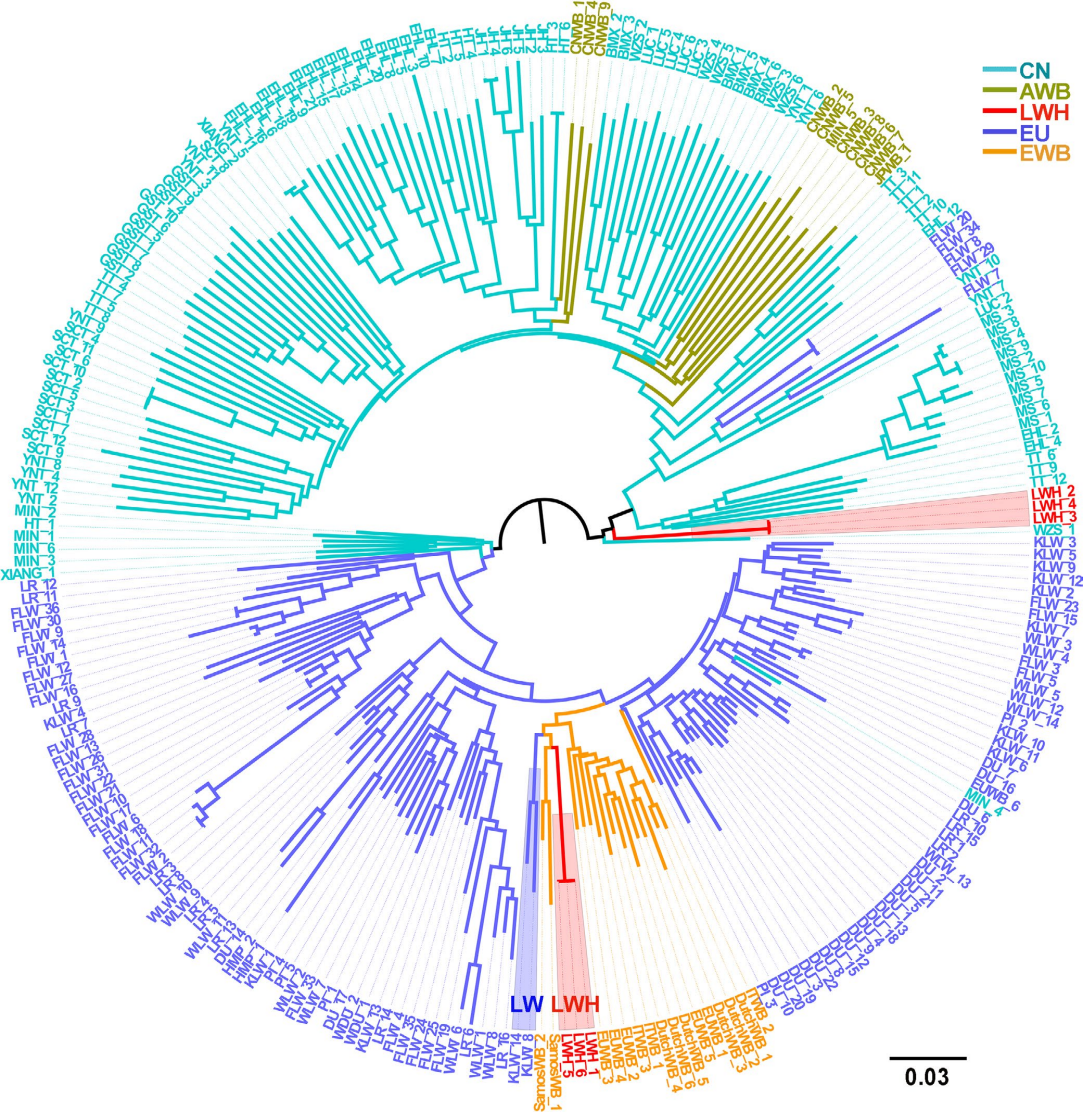
Neighbor‐joining (NJ) tree based on an identity‐by‐state matrix among 259 Eurasian pigs. NJ tree based on 13,899 qualified SNPs in the region of *GPC6*. CN, Chinese local pig breeds; AWB, Asian wild boar; EU, European modern pig breeds; and EWB, European wild boar

Integrative analyses of selection and introgression signatures showed that only one region (11:62642748–63096569) was overlapped for ROH, EigenGWAS, and *f_dM_*. The two top significant loci of EigenGWAS statistic locate at this introgressed region. Interestingly, this region resides in the long bone development‐related gene *GPC6* (11:62436143–63560908; Figure 6h). The haplotype heat map of the 2.4 Mb region (11:61964048–64362534) showed high selection and introgression signals, low level *dxy* between LWH2 and EU, and reduced π value in LWH2 (Figure 6c–f). The result indicated that most LWH2 individuals had the same haplotype, and were highly similar to those of European modern pigs. Haplotypes were also constructed for *GPC6* (21 SNPs), and the most frequent haplotype appeared 249 times in the 42 European pig breeds, including 218 LWH2 pigs, 22 LWH1 pigs, 3 BMEI pigs, 3 LW pigs, 3 USBK pigs, and 1 LR pig (Table S9). Most of the individuals in LWH1 (22/36) and LWH2 (218/300) carried this main haplotype, and this haplotype was not found in any other Chinese pig breeds, excepted for BMEI pigs which had been introgressed by European pigs reported in previous study (Huang et al., 2019). Then, we retrieved genomic DNA sequence of *GPC6* (13,899 qualified SNPs) from whole‐genome sequence data of 259 Eurasian pigs (Chen et al., 2020; Figure 7, Table S10). The IBS‐based NJ tree showed that three Laiwu pigs clustered with Large White pigs and European wild boars, supporting the introgression event at the *GPC6* loci.

### 3.6 Associations of introgressed haplotypes with body size of Laiwu pigs

**FIGURE 8:**
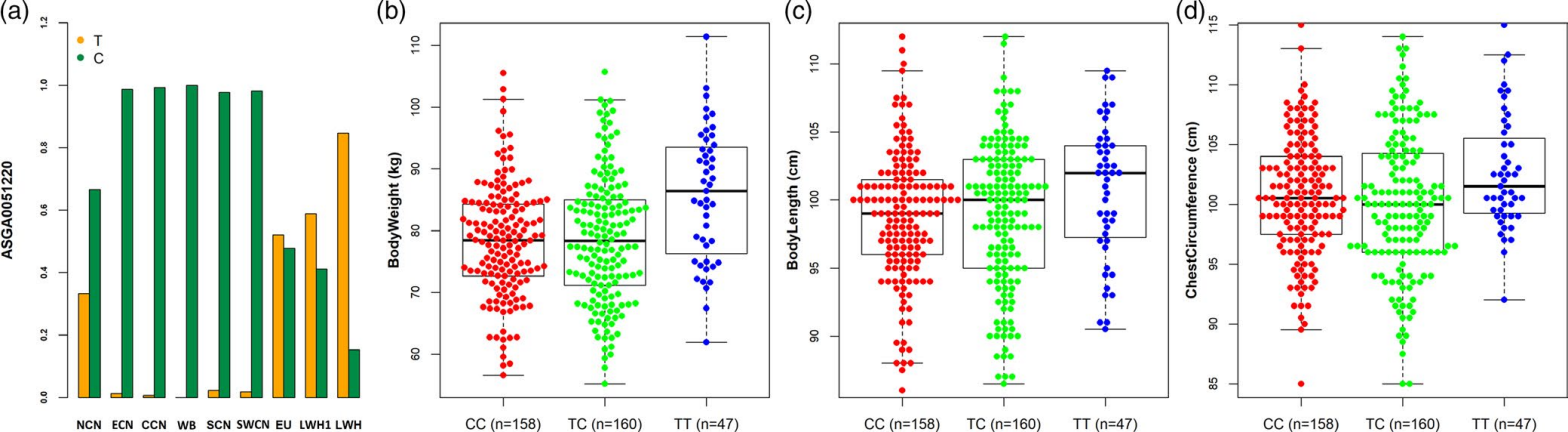
Association of ASGA0051220 with body size in Sujiang pigs. (a) Distribution of allele frequencies of ASGA0051220. (b) Body weight. (c) Body length. (d) Chest circumference

We identified three significant (*p* < .05) SNPs of *GPC6* by linear mixed model association analysis (Table 2, Table S11). We focused on the locus at 62755707 bp (SNP ASGA0051220) with significant selection (ROH and EigenGWAS) and introgression (*f_dM_*) signal. The allele *T* at ASGA0051220 was at high frequencies in Laiwu and European modern pigs but at very low frequencies in Chinese indigenous pigs (Figure 8a). The association analysis showed that ASGA0051220 significantly associated with body weight (*p* = .005) and chest circumference (*p* = .024), and individuals with the *TT* genotype had larger body size than those with genotypes *TC* and *CC* (Figure 8b–d). Our findings suggest that the introgressed European haplotypes have been preferentially selected for possibly improving growth performance, leading to their distribution at high frequencies in Laiwu pigs.

**TABLE 2:** Association analysis detected significant SNPs at GPC6 loci

SNP	Position	Linear mixed model		
		*p*_BW_	*p*_BL_	*p*_CC_
ASGA0051191	62508448	0.027*	0.144	0.077
ALGA0062892	62573661	0.038*	0.035*	0.047*
ASGA0051220	62755707	0.005**	0.108	0.024*

Note

*P*_WB_, *p* value of body weight; *p*_BL_, *p* value of body length; *p*_CC_, *p* value of chest circumference.

* Significant codes: .05.

** Significant codes: .01.

## 4 DISCUSSION

### 4.1 Genomic signature of introgression in Laiwu pigs

In this study, multiple evidences support that European haplotypes had been introgressed into Laiwu pigs. First, Laiwu pigs did not cluster with the Chinese major clade in the NJ tree and multiple Laiwu individuals displayed close genetic relationships with European modern pigs in the PCA plot. Moreover, the analyses of Admixture, *f3,* and D‐statistic clearly showed gene flow from European modern pigs to Laiwu pigs, and 13 Laiwu pigs had more than half genetic components of European origin. These findings were in agreement with the historical documents that European breeds were introduced to cross with local pigs in Shangdong province of China at three periods during the past century, including from 1920s to 1940s, from 1950s to 1970s, and from 1980s to 1990s (Wang et al., 2011). It is most likely that indiscriminate crossbreeding between Laiwu pigs and introduced European modern pigs occurred during these periods, leading to an average of 26.66% (Admixture, *qpAdm,* and *f_dM_*) genetic components from European modern pigs in the present conservation population of Laiwu pigs.

### 4.2 Effect of introgression events on the genetic diversity of Laiwu pigs

We analyzed the genetic diversity of two Laiwu populations, the present‐day and early‐day, in a world panel of pigs. Six statistics (Ho, He, Ne, *F*, *F*_ROH,_ and LD) collectively show that the current population has more abundant genetic diversity than the early‐day population. There findings were not expected, because the conservation population of Laiwu pigs was raised in a single farm without introduction of Laiwu pigs from other locations for the past decades. It is thus inevitable that inbreeding occurred in this population to a certain degree, resulting in a decreased level of genetic diversity. One reasonable explanation for this inconsistence is that recent admixture with exotic lineage increased the genetic diversity level in the current Laiwu population. This assumption is supported by the fact that Laiwu pigs have genomic signatures of admixture with European modern pigs, and the fact that the nucleus population of Lulai pig, a synthetic line derived from a cross between Laiwu and Large White, was also raised in this conservation farm for Laiwu pigs. It is likely that the two populations were not well managed, and occasional admixture between individuals from the two populations led to gene flow from Lulai pigs to Laiwu pigs and consequently increased genomic variability of the current conservation population of Laiwu pigs. This speculation was also confirmed by the result of nucleotide diversity (π) promotes 25.11% in the introgressed regions of current Laiwu pigs.

### 4.3 Sustainable conservation strategy for Laiwu pigs

Of the 233 Laiwu pigs tested in this study, 150 individuals (LWH2) had more than the average proportion (73.25%) of Chinese lineage, and the average proportion of LWH2 was 81%. We suggest that these 150 individuals should be included as the nucleus conservation population of Laiwu pigs, and the considerable level of genetic diversity has been retained from the current population. As revealed by the NJ tree, the 150 individuals pertain to ten families. The previous studies demonstrated that family rotational mating (F: R) could maintain 90% of genetic diversity in a livestock for more than 100 years (Lu, 2013) and effectively reduce inbreeding in populations (Honda, Nomura, & Mukai, 2004; Windig & Kaal, 2008). To reduce potential of inbreeding depression, we propose a boar‐mediated rotational crossing among the ten families and an equivalent number of boars and sows in each family generation by generation. Moreover, 80 Laiwu pigs including 43 LWH3 and 27 LWH4 pigs had more than half genetic components of Chinese origin. To maintain the genetic diversity of Laiwu pigs as much as possible, we suggest that LWH2 boars with low inbreeding (*F* and *F*_ROH_) values from different families mate with LWH3 and LWH4 sows to generate replacement gilts, providing additional genetic materials for the sustainable conservation of Laiwu pigs. As to 13 LWH5 individuals that had less than half genetic components of Chinese origin, it is better to remove these admixed (hybrid) individuals from the conservation population to maintain consanguinity purity of Laiwu pigs.

### 4.4 Genomic signatures of selection in Laiwu pigs

We performed ROH, iHS, and EigenGWAS to uncover genome‐wide footprints caused by natural and artificial selection in the 150 LWH2 pigs. We found few overlaps between the candidate regions identified by the three approaches. This is not surprising as there are differences in the statistics underlying each approach allowing to reveal the signatures of different types of selection across different timescales (Mastrangelo et al., 2020). Runs of homozygosity mainly results from population phenomena such as genetic drift, population bottleneck and inbreeding, the intensive artificial selection of superior animals as one factor has also reshaped the ROH patterns in various regions of the genome (Peripolli et al., 2017). The same as previous studies reported, some short and shared ROH islands were observed to be overlapped with regions under selection based the iHS testing, and showed significant positive correlation (Xu, Sun, et al., 2019; Zhang et al., 2015). The iHS test is especially powerful in detection of recent selection that has swept the selected allele to moderate frequencies but the selected allele has not yet been fixed (Tang, Thornton, & Stoneking, 2007). Conversely, the EigenGWAS statistic is similar as *F*_ST,_ which is more efficient in identifying loci fixed or closed to fixation for opposite alleles. Introgression and selection of European haplotypes at these loci in Laiwu pigs would lead to significant signals uncovered by EigenGWAS analyses. Additionally, given that the limited genome coverage (35K and 46K SNPs), it is possible that some important genome regions might not have been identified.

According to these factors and to further control the false‐positive rates, genomic regions of positive selection identified by at least two statistics were shortlisted. These detected loci could have experienced directional selection in Laiwu pigs, contributing to their breed characteristics, such as fecundity, desirable meat quality, except for the high intramuscular fat content. For example, we detected a list of genes putatively under selection that are functionally related to these breed features, such as *VIP*, *ESR1*, *AKAP12,* and *TGFBR3* for fecundity. *VIP* is an important regulating factor for testosterone biosynthesis and FSH secretion, playing a role in testicular aging (Lacombe et al., 2007). *ESR1* variants have been associated with litter sizes in pigs (Muñoz et al., 2007), and *ESR1* knockout mice develop obesity after sexual maturation (Ohlsson et al., 2000). Loss of the *AKAP12* results in prostatic hyperplasia and infertility in human and mice (Akakura, Huang, Nelson, Foster, & Gelman, 2008), and significantly affect feed conversion ratio in pigs (Messad, Louveau, Koffi, Gilbert, & Gondret, 2019). *TGFBR3* affects the growth and development of oocyte in pigs (Chermula et al., 2018). We also detected several candidate genes relating to growth and fat deposition traits. The genes *MYCT1*, *BARHL2*, *ZNF326*, *ABCC4,* and *GPC5* are identified as candidate genes for human body height in previous GWAS studies (Fox et al., 2007; Allen et al., 2010; Winkler et al., 2015; Wood et al., 2014). *SYNE1* knockout mice show growth retardation and decreased survival rates (Jianlin et al., 2010). *GDF6* plays an important role in formation of a diverse subset of skeletal joints (Clendenning & Mortlock, 2012). Mice lacking *SOX21* was with reduced growth, and *SOX21* variants may be a cause of nonendocrine short stature in human (Cheung, Okano, & Camper, 2017). Gain‐of‐function mutations in the phosphatidylserine synthase 1 (*PTDSS1*) gene cause Lenz‐Majewski hyperostotic dwarfism and developed a severe skeletal dysplasia (Piard et al., 2018). Mice lacking out *TP53INP1* were prone to obesity (Seillier et al., 2015). It is reported that the body weight, body length, reproductive performance, and lean meat percentage of Laiwu pigs have been improved from 1988 to 2007 (Wang et al., 2011). These improvements likely benefit by the positive selection and made the Laiwu pigs more competitive. These findings advance our understanding of the molecular mechanisms underlying the germplasm characteristics in Laiwu pigs.

### 4.5 Effect of introgression events on phenotypic traits in Laiwu pigs

Pigs were domesticated independently from European and Chinese wild boars nearly 10,000 years ago (Frantz et al., 2013), and different phenotypic characteristics harbor in Eurasian pigs. Over past decades, European modern breeds have been imported into China and admixed with many Chinese indigenous pig breeds (Wang et al., 2011). Our results confirmed the introgression has occurred from European modern pigs to Laiwu pigs genome. For introgressed regions, it is possible to identify several genes involved the growth‐related GO terms, considering that European modern pigs have been intensively selected for large body size, fast growth and lean pork yield.

We are particularly interested in the most significant selected and introgressed region containing the *GPC6* gene on SSC11. GPCs are a family of proteoglycans that are linked to the plasma membrane through a glycosylphosphatidylinositol anchor. GPCs regulate the signaling activity of various morphogens/growth factors, including Hedgehogs (Hhs; Wilson & Stoeckli, 2013) and bone morphogenetic proteins (Taneja‐Bageshwar & Gumienny, 2013). Loss‐of‐function mutations in the *GPC6* gene cause autosomal‐recessive omodysplasia in mice that is characterized by short stature, shortened limbs, and facial dysmorphism. Moreover, *GPC6* stimulates Hedgehog (Hh) signaling by binding to Hh and Patched 1 at the cilium and increasing the interaction of the receptor and ligand, and then promotes the growth of developing long bones (Capurro et al., 2017). In addition, the previous GWAS study identified that *GPC6* was a candidate gene correlated with the intramuscular fat of Duroc pigs (Ding et al., 2019). We explored genomic sequence data to confirm the introgression event at the *GPC6* locus, and further showed that the introgressed European *GPC6* haplotype was likely favorable for larger body size and weight and has been preferentially selected in Laiwu pigs, given that three SNPs at the *GPC6* locus were significantly associated with large body size in Sujiang pigs. Considering that the relatively small sample sizes of association analysis, and the tested population was not Laiwu pigs, additional studies are needed to definitively determine the role of *PGC6*. To our best knowledge, this study provides the first example of European introgressed haplotypes at a specific locus affecting growth performance in Chinese indigenous pigs, which advances our knowledge of the poorly understood effect of human medicated introgression on phenotypic traits in Chinese indigenous pigs.

## 5 COMPETING INTERESTS

None of the authors have any competing interests in the manuscript.

## AUTHOR CONTRIBUTIONS

J.R. designed the study and analyzed data. J.R. and X.W. wrote the paper. X.W., Z.Y., M.H., and J.T. performed statistical analyses. H.Z., L.Y., D.L., G.L., Y.J., Y.S., S.W., and P.X. collected samples and phenotypic data and performed genotyping experiments.

## Supporting information

Fig S1

Fig S2

Fig S3

Fig S4

Table S1

Table S2

Table S3

Table S4

Table S5

Table S6

Table S7

Table S8

Table S9

Table S10

Table S11

## Data Availability

The SNP genotype data are available in the Figshare Repository (https:// 10.6084/m9.figshare.12683321).
