# Supplementary figures and images for: Whole‐genome SNP markers reveal conservation status, signatures of selection, and introgression in Chinese Laiwu pigs
Source: Evol Appl. 2020 Sep 16;14(2):383–98. doi: 10.1111/eva.13124 (PMC7896721; doi:10.1111/eva.13124)

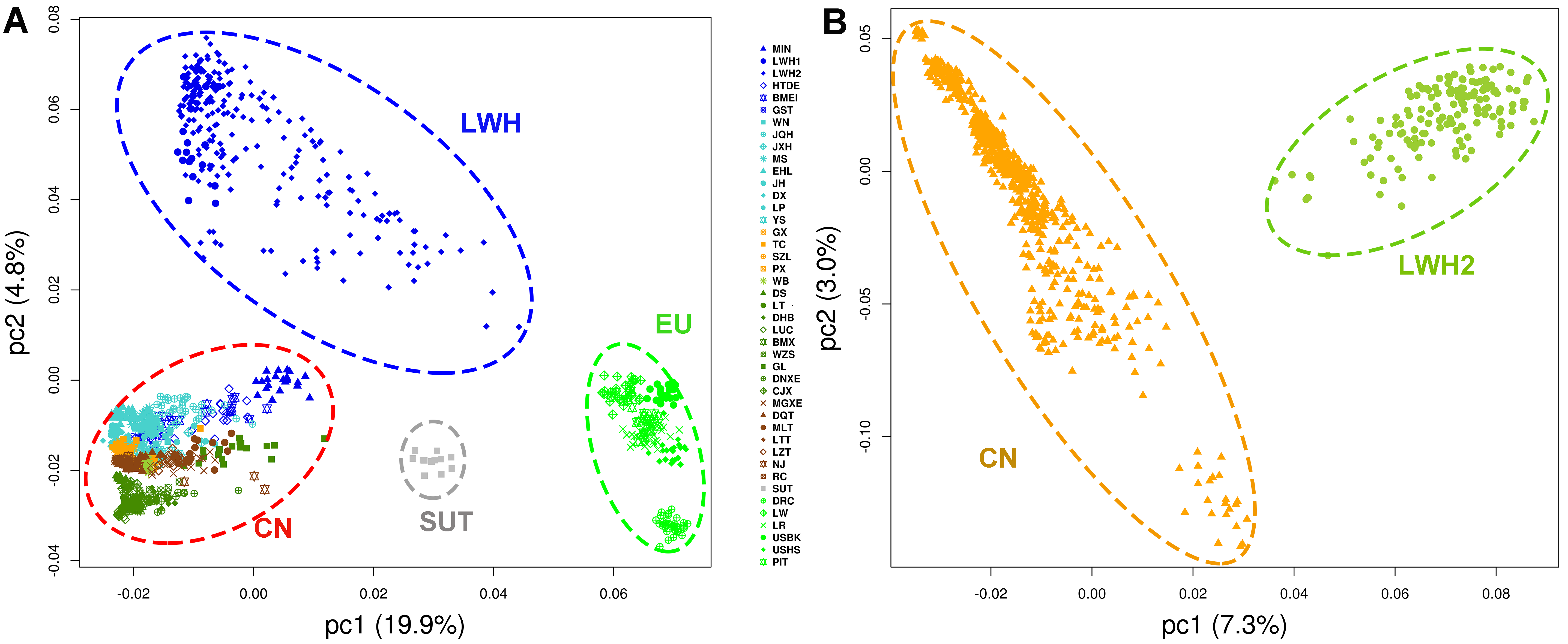

Supplement: Supplementary file 1 — Fig S1 [file EVA-14-383-s001.tif]

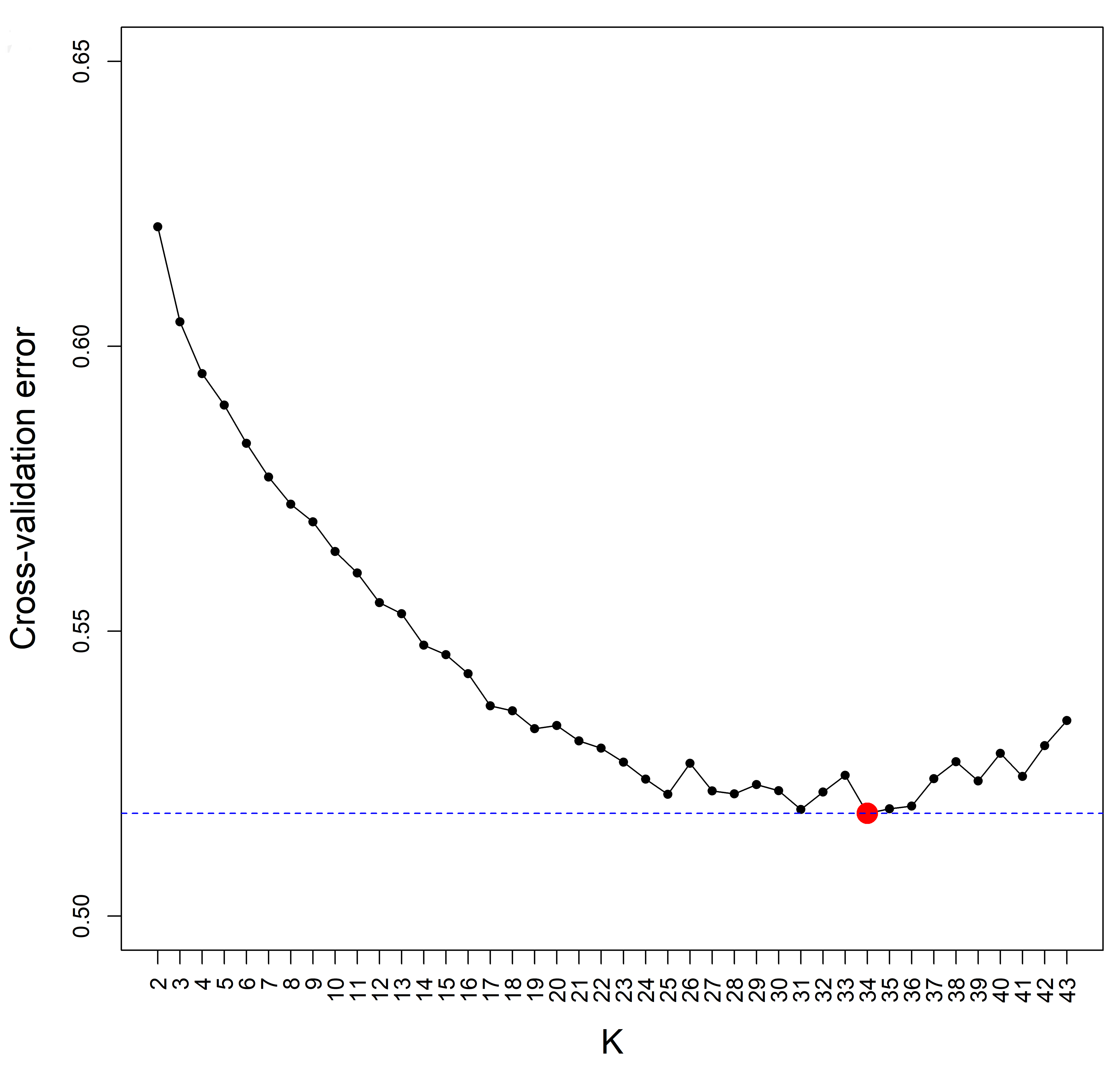

Supplement: Supplementary file 2 — Fig S2 [file EVA-14-383-s002.tif]

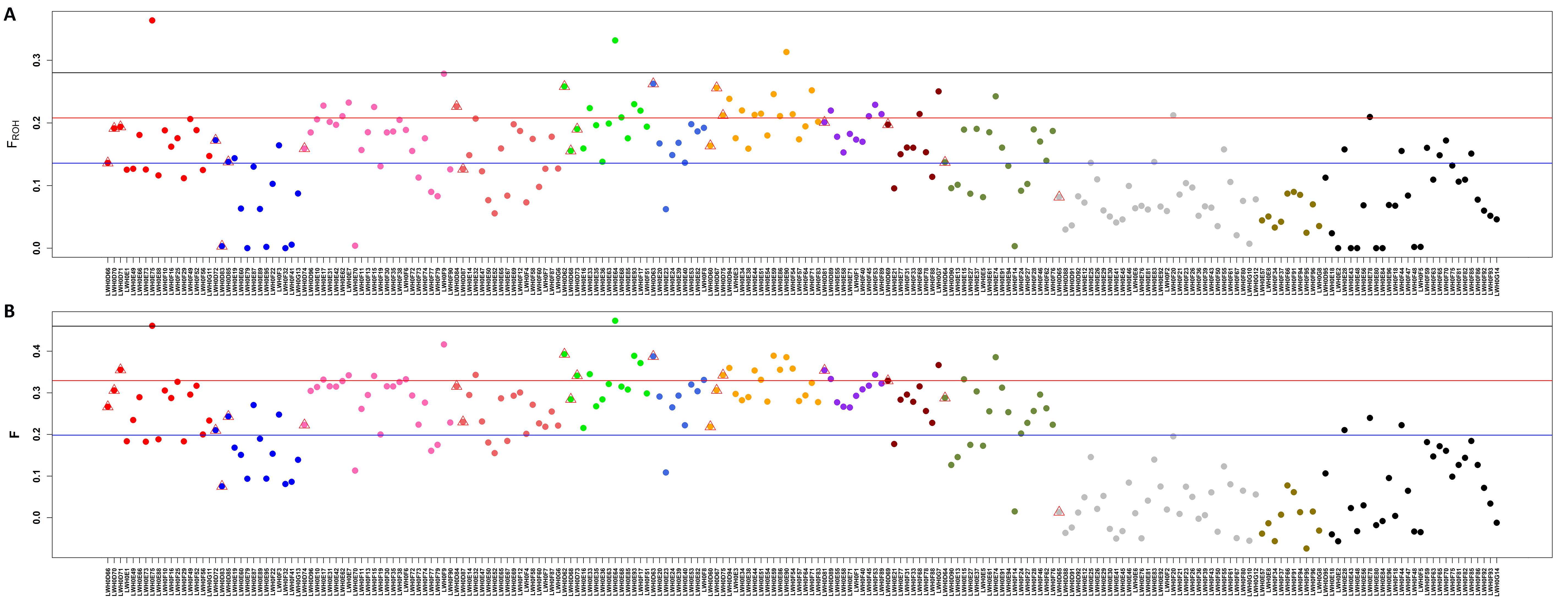

Supplement: Supplementary file 3 — Fig S3 [file EVA-14-383-s003.tif]

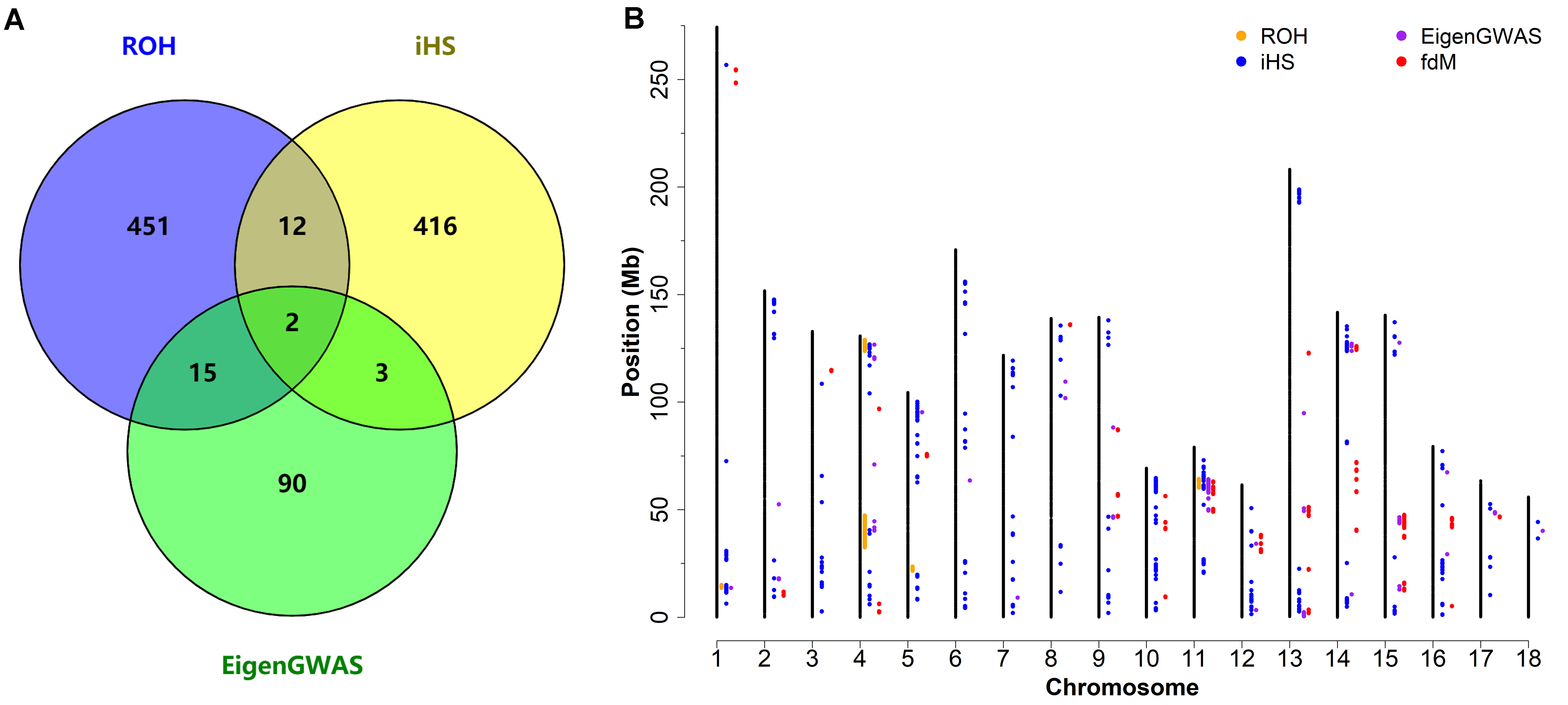

Supplement: Supplementary file 4 — Fig S4 [file EVA-14-383-s004.tif]
