# Supplementary material for: Whole‐genome SNP markers reveal conservation status, signatures of selection, and introgression in Chinese Laiwu pigs
Source: Evol Appl. 2020 Sep 16;14(2):383–98. doi: 10.1111/eva.13124 (PMC7896721; doi:10.1111/eva.13124)
Supplement: Supplementary file 5 — Table S1 [file EVA-14-383-s005.docx]

**Table S1 Genetic diversity of 43 pig breeds tested in this study**

| **Origin** | **Breed** | **Symbol** | **No** | **Ne** | **He** | **Ho** | **F** | **F_ROH_** | $r_{0.3}^{2}$ **(kb)** |
| --- | --- | --- | --- | --- | --- | --- | --- | --- | --- |
| NCN | Min | MIN | 22 | 107 | 0.172 | 0.181 | 0.214 | 0.136 | 209.002 |
|  | Laiwu | LWH1 | 18 | 120 | 0.148 | 0.152 | 0.308 | 0.185 | 195.114 |
|  | Laiwu | LWH2 | 150 | 271 | 0.235 | 0.244 | 0.270 | 0.164 | 79.307 |
|  | Laiwu | LWH | 233 | 241 | 0.337 | 0.349 | 0.187 | 0.133 | 66.138 |
|  | Hetaodaer | HTDE | 16 | 96 | 0.254 | 0.282 | 0.132 | 0.043 | 113.402 |
|  | Bamei | BMEI | 16 | 99 | 0.231 | 0.246 | 0.233 | 0.102 | 137.727 |
| ECN | Wannan | WN | 18 | 117 | 0.187 | 0.196 | 0.350 | 0.173 | 133.938 |
|  | Jiangquhai | JQH | 30 | 146 | 0.224 | 0.232 | 0.271 | 0.115 | 121.844 |
|  | Jiaxin | JXH | 30 | 158 | 0.172 | 0.166 | 0.464 | 0.226 | 130.958 |
|  | Meishan | MS | 20 | 107 | 0.175 | 0.147 | 0.503 | 0.293 | 170.426 |
|  | Erhualian | EHL | 32 | 188 | 0.187 | 0.187 | 0.350 | 0.077 | 55.174 |
|  | Jinhua | JH | 13 | 96 | 0.135 | 0.147 | 0.471 | 0.199 | 188.663 |
|  | Dongxiang | DX | 36 | 134 | 0.157 | 0.170 | 0.401 | 0.202 | 162.522 |
|  | Leping | LP | 20 | 128 | 0.210 | 0.216 | 0.279 | 0.102 | 84.795 |
|  | Yushan | YS | 36 | 151 | 0.201 | 0.211 | 0.309 | 0.148 | 123.610 |
| CCN | Ganxi | GX | 13 | 84 | 0.142 | 0.169 | 0.396 | 0.159 | 221.098 |
|  | Tongcheng | TC | 21 | 153 | 0.205 | 0.214 | 0.249 | 0.037 | 51.996 |
|  | Shaziling | SZL | 11 | 85 | 0.182 | 0.210 | 0.258 | 0.052 | 141.778 |
|  | Pingxiang | PX | 30 | 149 | 0.219 | 0.222 | 0.232 | 0.049 | 33.489 |
| WB | Wild Boar | WB | 21 | 169 | 0.221 | 0.214 | 0.242 | 0.032 | 8.593 |
| SCN | Dongshan | DS | 15 | 94 | 0.160 | 0.185 | 0.336 | 0.084 | 109.196 |
|  | Lantang | LT | 20 | 105 | 0.167 | 0.178 | 0.380 | 0.164 | 145.234 |
|  | Dahuabai | DHB | 16 | 85 | 0.182 | 0.210 | 0.295 | 0.162 | 221.793 |
|  | Luchuan | LUC | 18 | 135 | 0.157 | 0.166 | 0.402 | 0.084 | 71.596 |
|  | Bamaxiang | BMX | 16 | 118 | 0.196 | 0.206 | 0.281 | 0.052 | 55.356 |
|  | Wuzhishan | WZS | 16 | 128 | 0.228 | 0.236 | 0.191 | 0.013 | 36.723 |
|  | Diannanxiaoer | DNXE | 15 | 92 | 0.201 | 0.178 | 0.414 | 0.224 | 128.488 |
|  | Congjiangxiang | CJX | 16 | 100 | 0.163 | 0.183 | 0.355 | 0.154 | 174.903 |
| ESCN | Guanling | GL | 18 | 116 | 0.275 | 0.295 | 0.099 | 0.061 | 87.345 |
|  | Mingguangxiaoer | MGXE | 13 | 90 | 0.236 | 0.268 | 0.135 | 0.018 | 98.582 |
|  | Diqing Tebit | DQT | 19 | 137 | 0.213 | 0.218 | 0.241 | 0.045 | 35.468 |
|  | Gansu Tibet | GST | 21 | 139 | 0.201 | 0.215 | 0.271 | 0.060 | 70.194 |
|  | Milin Tebit | MLT | 16 | 129 | 0.261 | 0.267 | 0.148 | 0.045 | 50.851 |
|  | Litang Tebit | LTT | 16 | 121 | 0.191 | 0.182 | 0.347 | 0.117 | 32.128 |
|  | Linzhi Tebit | LZT | 29 | 135 | 0.216 | 0.231 | 0.240 | 0.074 | 81.711 |
|  | Neijiang | NJ | 16 | 109 | 0.191 | 0.202 | 0.317 | 0.098 | 111.813 |
|  | Rongchang | RC | 18 | 147 | 0.189 | 0.194 | 0.302 | 0.037 | 32.692 |
| Hybrid | Sutai | SUT | 12 | 91 | 0.307 | 0.323 | 0.107 | 0.131 | 228.007 |
| European | Duroc | DRC | 35 | 208 | 0.299 | 0.284 | 0.210 | 0.178 | 160.229 |
|  | Large White | LW | 35 | 214 | 0.363 | 0.353 | 0.028 | 0.086 | 104.841 |
|  | Landrace | LR | 35 | 207 | 0.350 | 0.333 | 0.081 | 0.117 | 111.795 |
|  | Berkshire | USBK | 20 | 148 | 0.258 | 0.262 | 0.284 | 0.179 | 205.819 |
|  | Hampshire | USHS | 20 | 142 | 0.297 | 0.303 | 0.174 | 0.138 | 181.356 |
|  | Pietrain | PIT | 20 | 136 | 0.323 | 0.333 | 0.082 | 0.152 | 196.481 |

NCN, North China; ECN, East China; CCN, Central China; WB, wild boar; SCN, South China; SWCN, Southwest China. Ne, effective population size; Ho, observed heterozygosity; He, expected heterozygosity; F, inbreeding coefficient; F_ROH_, runs of homozygosity as a percentage of the genome; $r_{0.3}^{2}$, extent of linkage disequilibrium when r^2^ = 0.3
