# Supplementary material for: Whole‐genome SNP markers reveal conservation status, signatures of selection, and introgression in Chinese Laiwu pigs
Source: Evol Appl. 2020 Sep 16;14(2):383–98. doi: 10.1111/eva.13124 (PMC7896721; doi:10.1111/eva.13124)
Supplement: Supplementary file 14 — Table S10 [file EVA-14-383-s014.docx]

**Table S9** Descriptive statistics of whole-genome sequence data of 259 Eurasian pigs.

| **Classification** | **Population** | **No.** | **Depth (×)** | **Accession number (No.)** |
| --- | --- | --- | --- | --- |
| Wild boar | Asian Wild Boar | 10 | 22.19 | PRJEB9922 (3)  PRJNA213179 (6)  PRJEB1683 (1) |
| East Chinese pigs | Erhualian | 21 | 28.17 | PRJNA550237 (2)  PRJNA213179 (6)  PRJNA488327 (13) |
|  | Jinhua | 6 | 26.24 | PRJNA398176 (6) |
|  | Meishan | 10 | 10.36 | PRJEB9922 (10) |
| South Chinese pigs | Bama Xiang | 6 | 27.13 | PRJNA213179 (6) |
|  | Luchuan | 6 | 26.37 | PRJNA213179 (6) |
|  | Wuzhishan | 6 | 26.09 | PRJNA213179 (6) |
|  | Xiang | 2 | 9.05 | PRJEB1683 (2) |
| Southwest Chinese pigs | Sichuan Tibetan | 12 | 26.78 | PRJNA550237 (6)  PRJNA213179 (6) |
|  | Xizang Tibetan | 12 | 25.95 | PRJNA213179 (6)  PRJNA550237 (6) |
|  | Yunnan Tibetan | 12 | 26.41 | PRJNA213179 (6)  PRJNA550237 (6) |
| North Chinese pigs | Gansu Tibetan | 10 | 26.66 | PRJNA550237 (6)  PRJNA213179 (4) |
|  | Laiwu | 6 | 26.14 | PRJNA213179 (6) |
|  | Min | 6 | 25.80 | PRJNA213179 (6) |
|  | Hetao | 6 | 24.40 | PRJNA213179 (6) |
| Wild boar | European Wild Boar | 17 | 12.90 | PRJEB9922 (17) |
| European Domestic pigs | French Large White | 36 | 22.03 | PRJNA550237 (36) |
|  | White Duroc | 2 | 36.81 | PRJNA550237 (2) |
|  | Duroc | 22 | 13.96 | PRJEB1683 (4)  PRJNA260763 (18) |
|  | Korea Large White | 14 | 16.18 | PRJNA260763 (14) |
|  | Dutch Large White | 14 | 9.40 | PRJEB1683 (13)  PRJEB9922 (1) |
|  | Hampshire | 2 | 10.81 | PRJEB1683 (2) |
|  | Landrance | 16 | 12.14 | PRJNA260763 (11)  PRJEB1683 (5) |
|  | Pietrain | 5 | 9.40 | PRJEB1683 (5) |
| Total |  | 259 |  |  |
